# SNRPD2 Is a Novel Substrate for the Ubiquitin Ligase Activity of the *Salmonella* Type III Secretion Effector SlrP

**DOI:** 10.3390/biology11101517

**Published:** 2022-10-17

**Authors:** Andrea Bullones-Bolaños, Juan Luis Araujo-Garrido, Jesús Fernández-García, Francisco Romero, Joaquín Bernal-Bayard, Francisco Ramos-Morales

**Affiliations:** 1 Departamento de Genética, Facultad de Biología, Universidad de Sevilla, Avda Reina Mercedes, 6, 41012 Sevilla, Spain; 2 Departamento de Microbiología, Facultad de Biología, Universidad de Sevilla, Avda Reina Mercedes, 6, 41012 Sevilla, Spain

**Keywords:** type III secretion, ubiquitination, SlrP, SNRPD2, RNA splicing

## Abstract

**Simple Summary:**

*Salmonella* is a genus of bacterial pathogens that can cause several diseases in humans and other animals. These bacteria can inject proteins known as effectors into animal cells through a secretion system. One of these effectors, SlrP, promotes the covalent addition of ubiquitin, a small eukaryotic protein, to specific host proteins, leading to an alteration of their stability or function. Here, we have performed a genetic screen to find new human targets of SlrP. In this way, we have identified SNRPD2, a core component of the spliceosome, the ribonucleoprotein complex that removes introns from eukaryotic pre-mRNA. SNRPD2 physically interacts with SlrP and is also a substrate of its ubiquitination activity. Lysines at positions 85 and 92 in SNRPD2 are among the residues that were ubiquitinated in the presence of SlrP. The identification of new host targets of *Salmonella* effectors contributes to a better understanding of the biological processes that are highjacked by these pathogens during infection, and can help in the design of future therapeutic strategies.

**Abstract:**

SlrP is a protein with E3 ubiquitin ligase activity that is translocated by *Salmonella enterica* serovar Typhimurium into eukaryotic host cells through a type III secretion system. A yeast two-hybrid screen was performed to find new human partners for this protein. Among the interacting proteins identified by this screen was SNRPD2, a core component of the spliceosome. In vitro ubiquitination assays demonstrated that SNRPD2 is a substrate for the catalytic activity of SlrP, but not for other members of the NEL family of E3 ubiquitin ligases, SspH1 and SspH2. The lysine residues modified by this activity were identified by mass spectrometry. The identification of a new ubiquitination target for SlrP is a relevant contribution to the understanding of the role of this *Salmonella* effector.

## 1. Introduction

*Salmonella* are Gram-negative bacteria that belong to the Enterobacteriaceae family. This genus includes thousands of serovars that can infect a wide variety of animals, causing different diseases, including gastroenteritis and typhoid fever, depending on the combination of serovar/host [1]. *Salmonella enterica* has two type III secretion systems (T3SS) encoded by *Salmonella* pathogenicity island 1 (SPI1) and SPI2, respectively, that are essential for the interaction with eukaryotic host cells [2,3,4]. These systems are present in many gram-negative symbionts and pathogens of animals and plants and are used to translocate effector proteins into the host cell cytoplasm [5]. The SPI1-encoded T3SS is expressed at the beginning of infection and is involved in a cell invasion mechanism that operates through the localized reorganization of actin filaments and the formation of membrane ruffles on the surface of host cells [6]. SPI2-encoded T3SS is expressed when *Salmonella* is located in its typical intracellular niche, the *Salmonella-*containing vacuole, in response to the acidic pH and the limitation of the nutrients characteristic of this compartment. Effectors translocated through the membrane of the *Salmonella*-containing vacuole manipulate the trafficking and maturation of this phagosome and promote the intracellular survival and replication of *Salmonella* [4]. Together, these systems secrete more than 40 effectors, and some of them are known to target key host functions, including host cell cytoskeleton, trafficking, death/survival pathways, NF-κB and MAPK signaling pathways, and adaptive immunity [7,8,9]. However, the functions and cellular targets of some effectors are not completely understood.

SlrP is a T3SS effector that was identified more than 20 years ago in *S. enterica* serovar Typhimurium as a host range factor that was necessary for complete virulence in mice, but not in calves [10]. The gene encoding this protein is located outside of SPI1 and SPI2, in a DNA region with features of horizontal acquisition. This gene is expressed under both SPI1 and SPI2-inducing conditions [11]. The expression of *slrP* is induced by the SPI1 regulators HilC, HilD, and RtsA [12,13]. In this context, LeuO and Lon are indirect regulators of the expression of *slrP* that act through HilD. Furthermore, the main direct activator of this gene under SPI2-inducing conditions is the two-component system PhoQ/PhoP [11]. SlrP is a protein of 765 amino acid residues with an N-terminal domain that directs translocation through T3SS [14]. This protein belongs to the LPX species-spanning family of effectors, a subtype of the leucine-rich repeat (LRR) protein superfamily, which also comprises the *Salmonella* effectors SspH1 and SspH2, the *Yersinia* effector YopM, and the *Shigella* IpaH effectors [15]. These proteins share a central LRR domain that contains several repeats of the LeuX_6_LeuX_2_Ile/LeuProX_3_Pro sequence motif that mediates binding to host target proteins. In addition, the *Salmonella* and *Shigella* members of this family of effectors have a C-terminal domain known as NEL, for “novel E3 ubiquitin ligase” [16]. So far, the only host ubiquitination target described for SlrP is thioredoxin-1 (Trx1), a redox regulatory protein whose activity is decreased by SlrP, leading to an increase in host cell death [17]. An additional binding partner of SlrP is the endoplasmic reticulum chaperone ERdj3 [18].

This work was undertaken to find new host targets for SlrP. We identify a list of potential binding partners for this effector and demonstrate that SNRPD2 is a substrate for its ubiquitin ligase activity.

## 2. Materials and Methods

### 2.1. Bacterial Strains, Yeast Strains, and Plasmids

The microbial strains and plasmids used in this study are described in Table 1. *Salmonella enterica* serovar Typhimurium strains were derived from the wild-type strain ATCC 14028. Transductional crosses using the phage P22 HT 105/1 *int201* [19] were used for the construction of *Salmonella* strains [20].

### 2.2. DNA Amplification with the Polymerase Chain Reaction and Sequencing

The amplification reactions were carried out on a T100 Thermal Cycler (Bio-Rad, Hercules, CA, USA) using Velocity DNA polymerase or MyTaq Red DNA polymerase (Bioline, London, UK) according to the supplier’s instructions. Oligonucleotides are described in Table 2. Constructs were sequenced with an automated DNA sequencer (Stab Vida, Oeiras, Portugal).

### 2.3. Plasmid Construction

Bacterial genes were amplified using the wild-type strain of *S. enterica* serovar Typhimurium 14028 as template, while eukaryotic genes were amplified from a human Jurkat cDNA library. To generate the plasmids pIZ3597 and pIZ3598, *sspH1* and *sspH2* inserts were PCR amplified using the primers P1-pQE80-sspH1-fw and P2-pQE80-sspH1-rv, or P1-pQE80-sspH2-fw and P2-pQE80-sspH2-rv, respectively. The pQE80L plasmid was amplified using the pQE80fw and pQE80rv primers. Then, both PCR products were ligated by Gibson assembly [28]. For the rest of the constructions, a classical cloning strategy based on enzymatic digestion was used.

### 2.4. Bacterial Culture

The standard culture medium for *S. enterica* and *Escherichia coli* was lysogeny broth (LB). Solid LB contained agar at 1.5% final concentration. Antibiotics were used at the following concentrations: kanamycin (Km), 50 μg/mL; ampicillin (Ap), 100 μg/mL.

### 2.5. Yeast Two-Hybrid Methods

A human Jurkat cDNA library constructed in fusion with the activation domain of Gal4 in pGAD1318 was screened. *S. cerevisiae* strain L40 was sequentially transformed with pIZ1628 (pLEX10-SlrP) and the library by the lithium acetate procedure [29]. The transformants were seeded in yeast drop-out medium lacking leucine, tryptophan, and histidine. The plates were incubated at 30 °C for 3 to 8 days and then colonies were patched on the same medium and replica-plated on Whatman 40 filters placed on drop-out medium lacking leucine and tryptophan to test the β-galactosidase activity [30]. Positive clones were rescued, tested for specificity using empty pLEX10, and sequenced with primer Gal4AD. 

### 2.6. Cell Culture, Lysis, and Transfection

HeLa (human epithelial; ECACC no. 93021013) and HEK293T (human embryonic kidney SV40 transformed; ECACC no. 12022001) cells were cultured in DMEM supplemented with 10% fetal calf serum. Amounts of 2 mM L-glutamine, 100 U/mL penicillin, and 100 μg/mL streptomycin were included in the culture medium. All cells were kept in a humidified atmosphere with 5% CO_2_ at 37 °C. For cell lysis, 2 × 10^7^ to 10^8^ cells per ml were incubated at 4 °C in NP40 buffer (10 mM Tris-HCl pH 7.4, 150 mM NaCl, 10% glycerol, 1% NP40, 1 mM PMSF, 1% protease inhibitor cocktail P8849 from Sigma-Aldrich) for 20 min. The extract was centrifuged at 20,000× *g* for 20 min and the supernatant was stored at −80 °C. For transient transfection assays, 2–5 × 10^6^ HeLa cells/assay were resuspended in 200 μL of 15 mM HEPES-buffered serum-containing medium, mixed with 50 μL of 210 mM NaCl containing 5–10 μg of plasmid DNA and electroporated using a BTX Electrocell Manipulator 600 set at 240 V, 950 μF, resistance = None. Cells were processed 24 h after electroporation. HEK293T cells were lipotransfected using the Xfect reagent (Takara Bio, Kusatsu, Japan) according to the manufacturer’s instructions, and processed 48 h after transfection.

### 2.7. GST and 6His Fusion Proteins, Electrophoresis, and Immunoblot

The expression of GST fusion proteins was induced by the addition of 1 mM isopropyl-β-D-thiogalactoside to *E. coli* BL21 (DE3) containing pGEX-4T-1, pGEX-4T-2, or their derivatives. For lysis, the bacteria were sonicated in NP40 buffer. The fusion proteins were isolated from bacterial lysates by affinity chromatography with glutathione-agarose beads (Sigma-Aldrich, Darmstadt, Germany). Then, 6His fusion proteins were produced after the addition of 1 mM isopropyl-β-D-thiogalactoside to *E. coli* XL1-Blue or M15/pREP4 containing derivatives of pQE30 or pQE80L, purified in Ni-NTA agarose beads (Sigma-Aldrich) and eluted with 300 mM imidazole in binding buffer (50 mM NaH_2_PO_4_, 300 mM NaCl). For some binding experiments, immobilized fusion proteins were incubated for 2 h with purified soluble proteins or cell lysates. The precipitates were washed six times in NP40 buffer followed by SDS-PAGE (polyacrylamide gel electrophoresis) and immunoblot. Anti-His (GE Healthcare, 1:3000), anti-FLAG M2 (Sigma-Aldrich, 1:5000), anti-SNRPD2 EPR16762 (abcam, 1:2000), and anti-HA-peroxidase 3F10 (Roche, 1:2000) were used as primary antibodies. Goat anti-mouse IRDye 800CW-conjugated or goat anti-rabbit IRDye 680RD-conjugated antibodies (LI-COR) were used as secondary antibodies. The bands were detected using the Odyssey Fc imaging system (LI-COR).

### 2.8. Mutagenesis

To generate point mutations in SNRPD2, pIZ3403 was used as a template for PCR amplification using primer pairs SNRPD2K85Afw/SNRPD2K85Arv, SNRPD2K92Afw/SNRPD2K92Arv, or SNRPD2-84-92Del-fw/SNRPD2-84-92Del-rv.

### 2.9. In Vitro Ubiquitination Assays

Ubiquitination reactions were carried out in a 20-μL mixture containing buffer A (25 mM Tris-HCl, pH 7.5, 50 mM NaCl, 5 mM ATP, 10 mM MgCl_2_, 0.1 mM DTT), 2 μg of HA-tagged ubiquitin, 0.25 μg of E1 (Boston Biochem, Cambridge, MA, USA) and 1 μg of E2 (human recombinant UbcH5b from Boston Biochem) in the presence or absence of 1 μg of 6His-SlrP, GST, or GST-SNRPD2. Reactions were incubated at 37 °C for 1 h with shaking and stopped by adding an equal volume of Laemmli sample buffer containing 100 mM DTT and boiling. Some reactions were carried out with GST fusion proteins bound to glutathione-agarose beads and the beads were washed five times with NP40 buffer before boiling in Laemmli sample buffer with 100 mM DTT.

### 2.10. Analysis of SNRPD2 Ubiquitination Sites by MALDI-MS(/MS)

Ubiquitinated and non-ubiquitinated purified proteins were loaded on a 10% acrylamide gel and Coomassie stained with Instant Blue (Abcam, Cambridge, UK). Bands of the ubiquitinated protein with a higher molecular weight compared with those of the nonubiquitinated form were excised and analyzed in the BIO-MS mass spectrometry facility of the Universidad Pablo de Olavide.

Acrylamide plugs were destained with NH_4_HCO_3_ and acetonitrile. Cysteine residues were reduced with DTT and alkylated with iodoacetamide. The protein was digested with trypsin. After digestion, the peptides were extracted with acetonitrile, acidified, and desalted in a C18 column. Mass spectra were obtained with a MALDI-TOF Ultraflextreme (Bruker, Billerica, MA, USA) in the TOF-TOF mode. The obtained fingerprint spectrum was compared against the simulated trypsin digestion of SNRPD2 sequence considering cysteine carbamidomethylation as a fixed modification and methionine oxidation and lysine ubiquitination as possible modifications. Peptides with a predicted ubiquitinated lysine were fragmented to confirm the presence of the modification.

### 2.11. Quantification of Protein Bands and Statistics

Fluorescent or luminescent signals from immunoblots detected with the Odyssey Fc imaging system (LI-COR) were quantified using Image Studio Lite software (LI-COR). SNRPD2 signals were corrected using β-actin as internal loading control. Means and standard deviations were calculated and one-way ANOVA with Dunnett’s post-hoc test or a Student’s t test were used to evaluate if the differences between conditions were significant.

## 3. Results

### 3.1. Identification of Mammalian Binding Partners for Salmonella SlrP through a Yeast Two-Hybrid Screen

**Figure 1:**
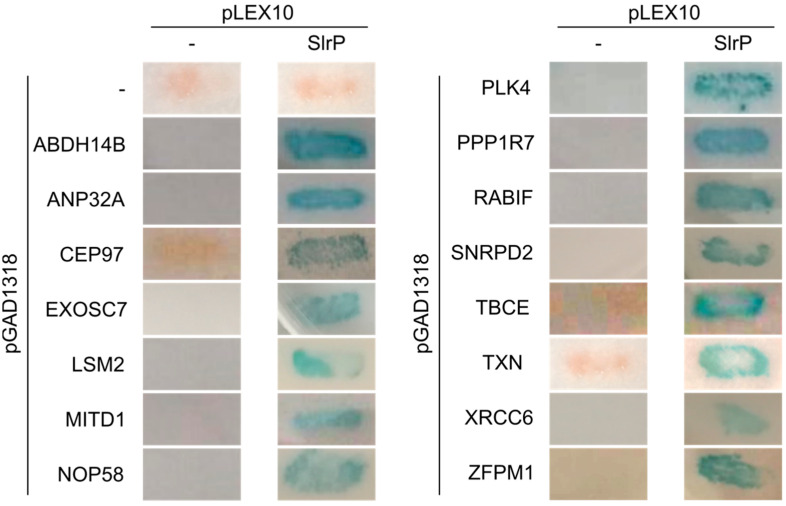
Analysis of interactions of SlrP with human proteins in the yeast two-hybrid system. Derivatives of plasmid pGAD1318 expressing the indicated proteins (or C-terminal fragments of these proteins) were introduced in yeast strain L40 together with pLEX10 or pLEX10-SlrP. The interaction between SlrP and human proteins is shown by the detection of blue color in the presence of X-Gal after a β-galactosidase filter assay. Empty vectors were used as negative controls.

To find new interacting partners for the *Salmonella* T3SS effector SlrP, we carried out a yeast two-hybrid screen using pLEX10 as bait vector and a human cDNA library that was prepared using the vector pGAD1318. The screening was carried out in strain L40 of *S. cerevisiae*, which carries two reporter genes to detect the interactions: *HIS3*, which complements an auxotrophy, and *lacZ*, which codes for the enzyme β-galactosidase. A total of 3 × 10^6^ clones were screened and 1400 colonies were able to grow in synthetic medium lacking histidine that was used to select for the interactions. Furthermore, 588 of these clones also showed β-galactosidase activity. The plasmids recovered from these clones were subjected to PCR using primers specific for thioredoxin cDNA, since thioredoxin is an SlrP partner identified in a previous screen. Indeed, 220 candidates were identified as cDNA encoding thioredoxin. The sequencing of some of the remaining candidates revealed that most of them corresponded to cDNA for SNRPD2 (small nuclear ribonucleoprotein D2), which plays a role in pre-mRNA processing. Finally, 30 new potential partners for SlrP were identified by a combination of DNA sequencing and PCR amplification. A clone of each candidate was reintroduced in yeasts containing pLEX10-SlrP or the empty vector to test the specificity of the interactions. A specific interaction in the two-hybrid system was observed for 14 candidates in addition to thioredoxin (Figure 1 and Table 3).

### 3.2. Confirmation of the Interaction of SlrP with SNRPD2

**Figure 2:**
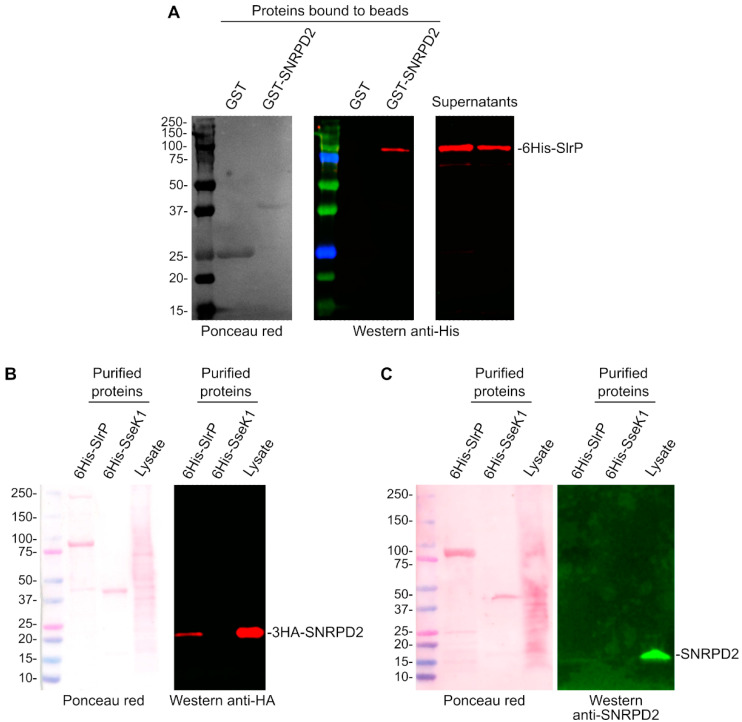
Confirmation of the interaction of SNRPD2 with SlrP. (**A**) 6His-SlrP was incubated with GST or GST-SNRPD2 bound to glutathione-agarose beads. Copurification of SlrP with SNRPD2 was detected by immunoblot with anti-His antibodies. (**B**) 6His-SlrP or 6His-SseK1 bound to Ni-NTA agarose beads were incubated with a cell lysate obtained from HeLa cells expressing 3HA-SNRPD2. Copurification of SNRPD2 with SlrP was detected by immunoblot with anti-HA antibodies. Ponceau S red staining was used as loading control. (**C**) 6His-SlrP or 6His-SseK1 bound to Ni-NTA agarose beads were incubated with a cell lysate obtained from HeLa cells. Copurification of SNRPD2 with SlrP was detected by immunoblot with anti-SNRPD2 antibodies. Ponceau S red staining was used as loading control. Sizes of molecular weight markers are shown in kDa. Results are representative of two independent experiments.

Most of the clones detected in the two-hybrid screen described in the previous section expressed the host protein SNRPD2. In addition, several independent clones encoding this protein were isolated (clones of different sizes as indicated in Table 3). Therefore, we decided to focus on studying the interaction of this protein with SlrP. Two independent approaches were used to confirm the interaction. For the first approach, purified 6His-SlrP was incubated with GST or GST-SNRPD2 bound to glutathione-agarose beads for an hour. After extensive washing with NP40 buffer, the copurification of SlrP with SNRPD2 was demonstrated by Western blotting with anti-His antibodies. Copurification was not observed with GST alone, which was used as a control (Figure 2A). For a second approach, epithelial human HeLa cells were transfected with a plasmid expressing 3HA-SNRPD2, and protein extracts from these cells were incubated with 6His-SlrP bound to Ni-NTA agarose beads. The copurification of 6His-SlrP and 3HA-SNRPD2 was shown by Western blot using anti-HA antibodies. SNRPD2 was not copurified with 6His-SseK1, an unrelated *Salmonella* effector that was used as a control (Figure 2B). The copurification of 6His-SlrP with endogenous SNRPD2 was also observed (Figure 2C). In this case, a weaker band is detected, probably due to the lower sensitivity of the anti-SNRPD2 antibody.

### 3.3. SNRPD2 Is a Target of the E3 Ubiquitin Ligase Activity of SlrP

**Figure 3:**
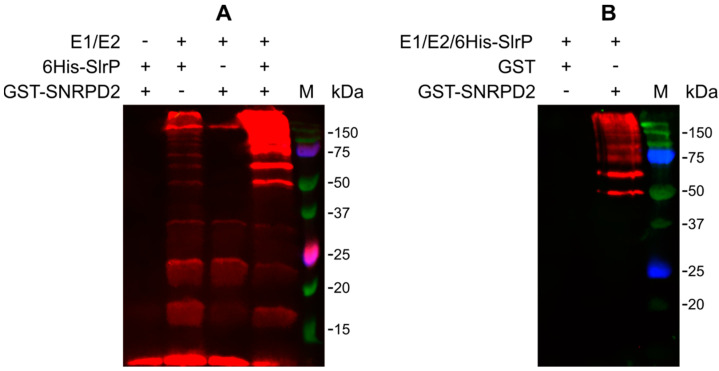
In vitro ubiquitination of SNRPD2 catalyzed by SlrP. (**A**) Ubiquitination reactions carried out with HA-ubiquitin in the presence (+) or absence (−) of E1, E2, 6His-SlrP, and GST-SNRPD2, were submitted to immunoblot analysis with anti-HA monoclonal antibodies. (**B**) Ubiquitination reactions were carried out with GST or GST-SNRPD2 bound to glutathione-agarose beads, washed and subjected to immunoblot analysis with anti-HA monoclonal antibodies. Results shown are representative of three independent experiments.

The main objective of this study was the detection of new targets for the E3 ubiquitin ligase activity of SlrP. As an interacting partner of SlrP, SNRPD2 may also be a substrate of its catalytic activity. To test this hypothesis, in vitro reactions were performed mixing HA-ubiquitin, E1, E2, 6His-SlrP in the presence or absence of GST-SNRPD2. As seen in Figure 3A, intense signals that may correspond to the ubiquitinated forms of GST-SNRPD2 were observed. Less intense bands were also detected in the absence of SNRPD2. These are probably polyubiquitinated forms of ubiquitin induced by SlrP [17]. To confirm these results, new ubiquitination reactions were carried out with GST-SNRPD2 or GST bound to glutathione-agarose beads. The beads were then extensively washed before ubiquitination and analyzed by immunoblotting with anti-HA antibodies. As seen in Figure 3B, with this protocol, ubiquitinated bands were only observed in the reactions with GST-SNRPD2 but not with GST alone. The ubiquitination ladder obtained suggests that several ubiquitin adducts can be attached to SNRPD2 to generate polyubiquitinated forms of this substrate.

### 3.4. Specificity of the Interaction and Ubiquitination of SNRPD2

**Figure 4:**
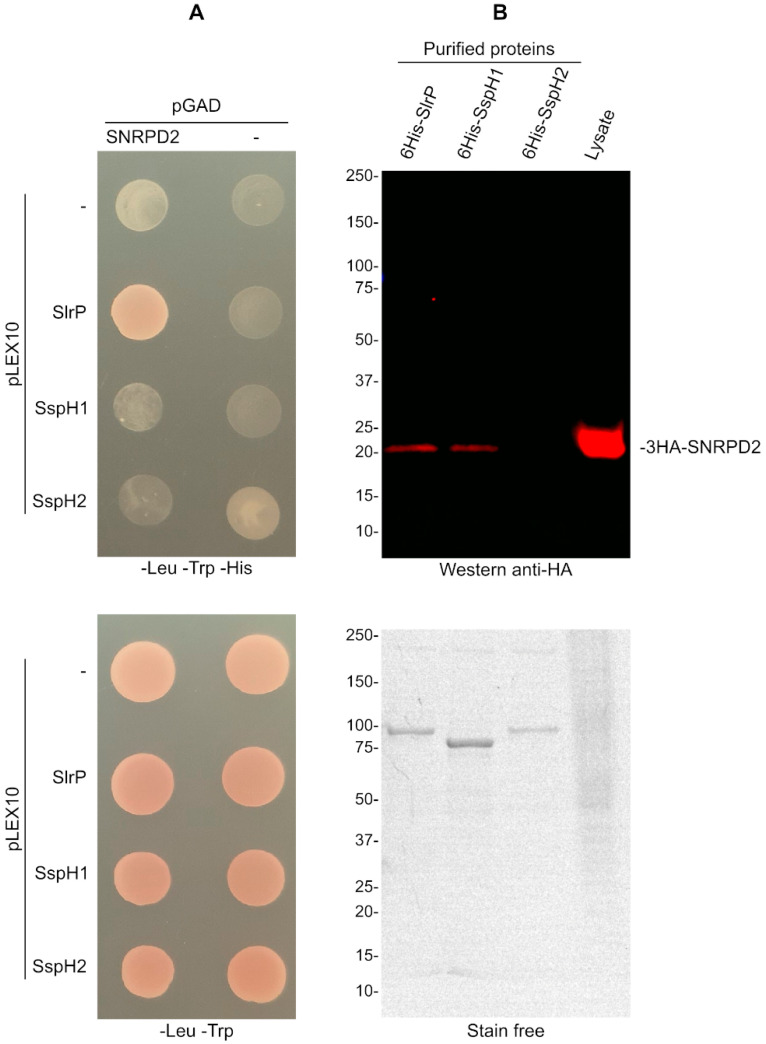
Specificity of the interaction of SNRPD2. (**A**) Derivatives of pLEX10 and pGAD1318 were introduced in yeast strain L40 by transformation. Transformants were selected in media lacking tryptophan and leucine. The interactions between the indicated effectors and SNRPD2 are analyzed by growth in the absence of histidine. (**B**) 6His-SlrP, 6His-SspH1 or 6His-SspH2 bound to Ni-NTA agarose beads were incubated with a cell lysate obtained from HeLa cells expressing 3HA-SNRPD2. Copurification of SNRPD2 with *Salmonella* effectors was detected by immunoblot with anti-HA antibodies. Stain-free total protein staining was used as loading control. Sizes of molecular weight markers are shown in kDa. Results are representative of two independent experiments.

SspH1 and SspH2 are two effectors of *Salmonella* that, together with SlrP, belong to the same family of NEL E3 ubiquitin ligases. Therefore, we decided to test whether these effectors were also able to interact and/or ubiquitinate SNRPD2. Interactions were studied using the yeast two-hybrid system and pull-down experiments. As seen in Figure 4A, only SlrP, but not SspH1 or SspH2, was able to interact with SNRPD2 in vivo in the two-hybrid system, although interaction was also observed between 6His-SspH1 and 3HA-SNRPD2 in an in vitro pull-down assay (Figure 4B).

**Figure 5:**
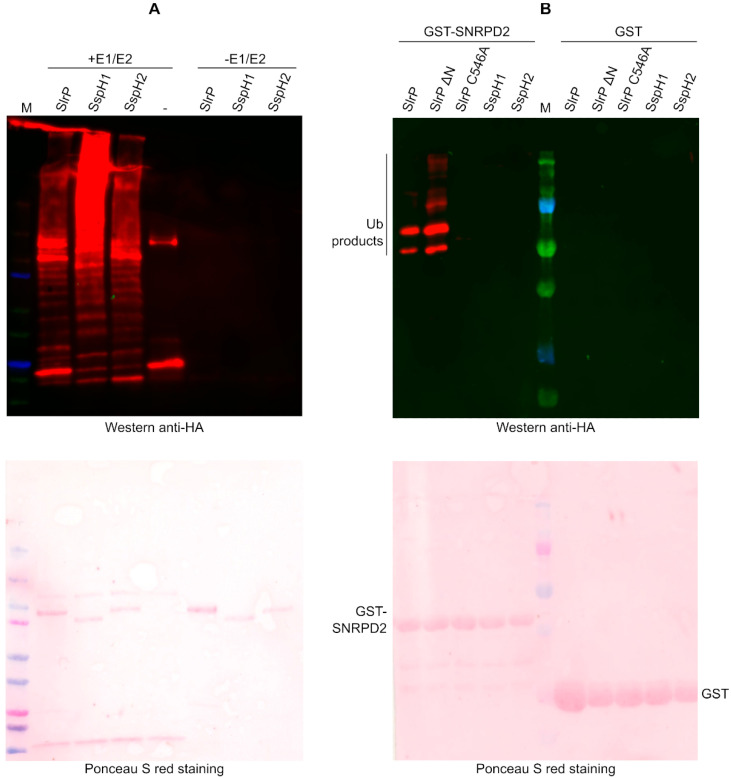
Specificity of the ubiquitination of SNRPD2. (**A**) The activity of 6His-tagged SlrP, SspH1 and SspH2 was tested with HA-ubiquitin in the presence or in the absence of E1, and E2. SlrP: 6His-SlrP, SspH1: 6His-SspH1, SspH2: 6His-SspH2, -: no E3 ligase effector added. (**B**) Ubiquitination of GST-SNRPD2 bound to glutathione-agarose beads was tested in the presence of HA-ubiquitin, E1, E2, and a *Salmonella* effector fused to 6His: wild-type SlrP, SlrP ∆N (lacking 139 N-terminal residues), SlrP C546A mutant, wild-type SspH1 or wild-type SspH2. GST was used as negative control. Beads were washed before the immunoblot analysis. Ponceau S red staining is shown in the lower panel. M, molecular weight markers (size of bands in kDa: 250, 150, 100, 75, 50, 37, 25, 20).

To study the ability of these effectors to specifically ubiquitinate SNRPD2, first we checked that the 6His-tagged forms of the three effectors were active as E3 ubiquitin ligases, as in the presence of E1 and E2 they were able to induce the polyubiquitination of ubiquitin (Figure 5A). Importantly, the in vitro ubiquitination experiment shown in Figure 5B indicates that SNRPD2 is not a substrate for the activity of SspH1 or SspH2. This experiment also shows that the ubiquitination activity of SlrP on SNRPD2 is lost when the catalytically important residue Cys^546^ is changed into Ala [17].

### 3.5. Analysis of SNRPD2 Ubiquitination by Mass Spectrometry and Mutagenesis

**Figure 6:**
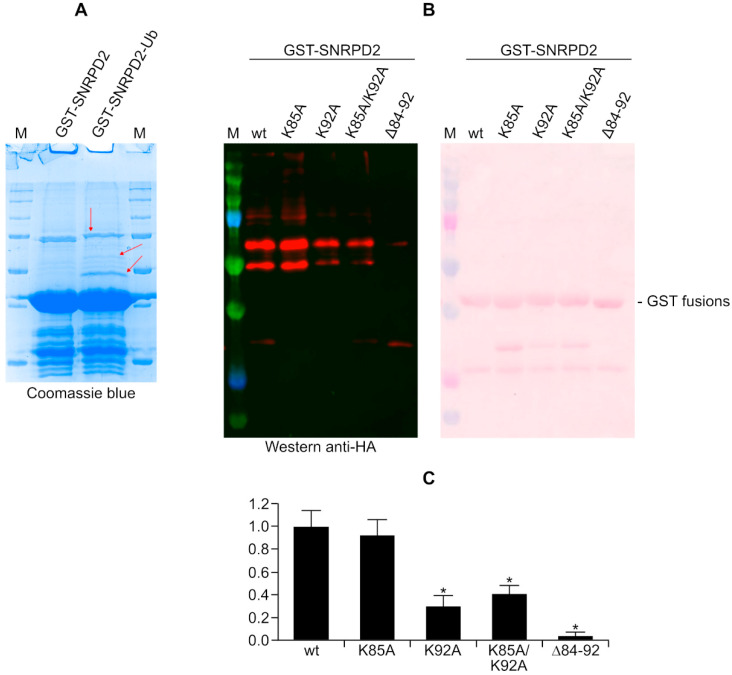
Identification of ubiquitinated residues in SNRPD2. (**A**) SDS-PAGE analysis of GST-SNRPD2 and GST-SNRPD2 subjected to the in vitro ubiquitination assay (GST-SNRPD2-Ub). The gel was stained with Coomassie blue and bands indicated by red arrows were cut for analyses by mass spectrometry. (**B**) Ubiquitination reactions catalyzed by SlrP on GST-SNRPD2 carried out in the presence of HA-ubiquitin were analyzed by Western blot with anti-HA antibodies. Different versions of GST-SNRPD2 used are indicated: wild-type (wt), point mutants K85A, K92A and K85A/K92A and deletion mutant Δ84-92. M, molecular weight markers (size of bands in kDa: 250, 150, 100, 75, 50, 37, 25, 20). (**C**) Quantification of ubiquitinated bands from three independent experiments. Means and standard deviations are represented relative to the wild-type values that were set to 1. * *p* < 0.05, for comparison with the wild type using ANOVA with Dunnett’s post-hoc test.

Next, we made efforts to identify residues in SNRPD2 whose ubiquitination is catalyzed by SlrP. The untreated GST-SNRPD2 protein was compared with the same protein after the in vitro ubiquitination assay by SDS-PAGE and Coomassie staining (Figure 6A). Some putative ubiquitinated bands were cut from gels and analyzed by mass spectrometry. As a result of this analysis, lysines at positions 85 and 92 in the SNRPD2 sequence were identified as ubiquitinated residues. These two lysines are contained in the tryptic peptide 83-92: GKKKSKPVNK. To confirm the relevance of these residues in the ubiquitination process under study, we generated point mutants where these lysines were mutated to alanines (mutants K85A and K92A) and a double mutant (K85A K92A). Furthermore, since the peptide that contains these lysines contains five lysines, we also generated a deletion mutant that lacks all of these residues (SNRPD2 mutant ∆84-92). These mutant versions of SNRPD2, together with the wild-type protein, were tested as SlrP substrates in an in vitro ubiquitination assay. As seen in Figure 6B,C, a partial but significant reduction in ubiquitination was detected for the K92A mutant and the double mutant K85A K92A. The reduction was more dramatic in the deletion mutant that lacks five lysines.

### 3.6. Lack of Effect of SlrP on SNRPD2 Levels

**Figure 7:**
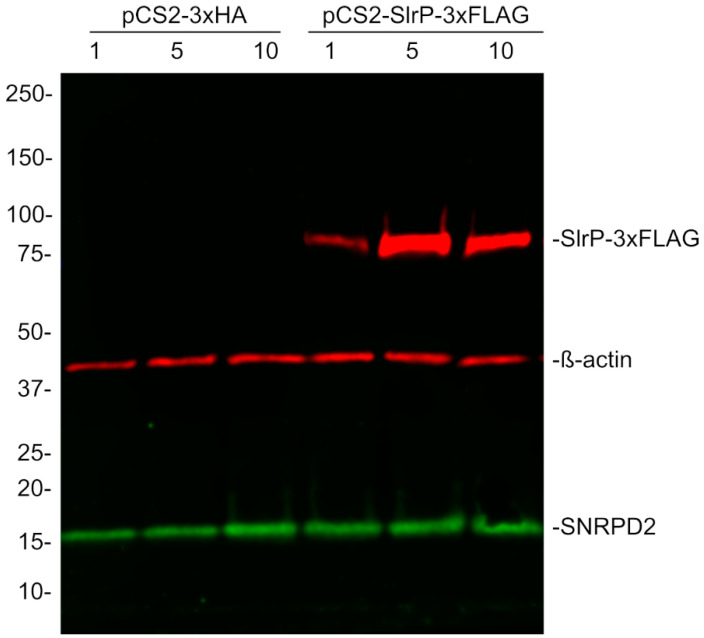
Stability of SNRPD2 in the presence of SlrP. HEK293T cells were transfected with 1, 5 or 10 μg of plasmids pCS2 or pIZ1720 (pCS2-SlrP-3xFLAG), as indicated. The level of SNRPD2 was detected by Western blot with anti-SNRPD2 antibodies. Expression of SlrP-3xFLAG was detected with anti-FLAG antibodies. Anti-β-actin antibodies were used as loading control. Sizes of molecular weight markers are shown in kDa. Results are representative of three independent experiments.

In many cases, ubiquitination is a signal for proteasome-dependent degradation. Therefore, we decided to investigate whether the levels of SNRPD2 were altered by the presence of SlrP. Subsequently, human HEK293T cells were transfected with different amounts of plasmid pIZ1720, a derivative of plasmid pCS2 encoding SlrP-3xFLAG, or an empty vector. As seen in Figure 7, the presence of SlrP did not change the amount of SNRPD2 detected with anti-SNRPD2 antibodies. The quantification of bands from three independent experiments indicated that the ratio between the amount of SNRPD2 in SlrP transfected cells and vector transfected cells is not significantly different from 1 (mean 1.33, standard deviation 0.48, *p* = 0.15).

## 4. Discussion

A previous yeast two-hybrid screen carried out in our laboratory identified two binding partners for the T3SS effector SlrP that was expressed from the plasmid pGBT10 [17,18]. In order to find new interacting partners, we carried out a screen using pLEX10 as vector. Unlike pGBT10, which contains a truncated ADH1 promoter, this vector contains the full-length promoter, allowing higher expression of the bait fusion protein and improving screen sensitivity [31]. In fact, using this vector, we were able to identify 14 additional candidate partners, suggesting that many more interacting partners may be revealed by different experimental approaches.

The vast majority of clones isolated in this two-hybrid screen expressed either thioredoxin or SNRPD2. Our group had previously studied the interaction of SlrP with thioredoxin [17,27]. Therefore, we decided to focus this work on the SlrP-SNRPD2 interaction. This interaction was confirmed using two additional independent methods.

SNRPD2, small nuclear ribonucleoprotein (snRNP) SmD2, is a protein of 118 amino acids that is a component of the spliceosome, a complex consisting of five snRNPs and numerous associated proteins known as splicing factors [32]. SNRPD2 is one of the 141 proteins considered core components of the human spliceosome based on their high abundance or function [33,34]. It is found in the precatalytic spliceosome B complex, the activated spliceosome C complexes, and the minor U12 spliceosome [35,36,37]. This protein plays a role in the appropriate cohesion of sister chromatids and cell proliferation [38] and in the nuclear retention of lncRNAs [39]. Its expression has also been proposed as a marker of prognosis in hepatocellular carcinoma [40,41] and as a factor that bridges mild cognitive impairment and Alzheimer’s disease [42].

Interestingly, the two-hybrid screen also revealed the interaction of SlrP with LSM2, another core component of the U4/U6-U5 tri-snRNP complex, which is involved in the assembly of spliceosomes and is a component of the precatalytic spliceosome B complex [43]. The fact that SlrP can interact and even ubiquitinate some components of the spliceosome suggests that this is a new host process that may be targeted by *S. enterica* serovar Typhimurium. Interestingly, there are some precedents in this regard. In fact, infections of human primary macrophages with *Salmonella* or *Listeria* induce differential isoform usage for many genes [44], notably genes involved in immune responses. These alternative splicing changes have been suggested to be critical for regulating innate immune gene expression and controlling infection outcomes in macrophages [45]. In a previous work, infection with *Salmonella* or *Yersinia* was also shown to amplify the alternative splicing of pre-mRNA for the HLA-B27 class I major histocompatibility complex that leads to the generation of a cell-free soluble protein isoform [46]. The signal inducing this event was not identified, but appeared to be specific, since incubation with IFNγ or lipopolysaccharide did not produce the same effect. Interestingly, another member of the NEL family of E3 ubiquitin ligases, IpaH9.8 from *Shigella flexneri*, targets the splicing factor U2AF35 (also known as U2AF1) [47]. Another recent study that aimed to reveal the interactome of *Salmonella* T3SS effectors showed that SlrP may interact with various host partners involved in RNA processing [48]. The results obtained in the present work suggest that the effector SlrP may be one factor that allows *Salmonella* to manipulate the host splicing system.

The ubiquitination of SNRPD2 catalyzed by SlrP was characterized at the molecular level by mass spectrometry. Lysines 85 and 92 were identified as ubiquitinated and mutagenesis analysis confirmed that residue 92 was indeed a preferential target for the catalytic activity of SlrP. However, the mutation of both residues did not completely abolish ubiquitination. This is not surprising since there are examples of other E3 ligases with low lysine specificity that target a large ubiquitination zone, where several lysines can be ubiquitinated [49]. The mutation of preferred ubiquitination sites does not prevent these E3s from ubiquitinating another site on the same substrate [50]. Lysines represent 5.7% of the total human proteome [51] but SNRPD2 has higher contents with 14.4% of this amino acid. Furthermore, the two residues identified as ubiquitinated are in a 20 residue stretch with 50% lysines that may represent the preferred ubiquitination zone targeted by SlrP in this protein.

The expression of SlrP in HEK293T cells did not appear to trigger SNRPD2 degradation. Although there is the possibility that the conditions used in our experiments were not sensitive enough to detect a low level of degradation, this result suggests that ubiquitination in this case may have a non-proteolytic function. In fact, there are many examples of the involvement of mono- and polyubiquitination in functions such as protein kinase activation, DNA repair, vesicle trafficking or transcription factor activity regulation, without affecting the stability of the target proteins [52]. A plausible consequence of the ubiquitination of SNRPD2 would be the interference with a proper spliceosome assembly. SNRPD2 (also known as SmD2) is one of seven core Sm proteins (B/B’, D1, D2, D3, E, F, and G). The proposed assembly of these proteins suggests a heptamer model that form a ring that interacts with the Sm site in U1, U2, U4 and U5 small nuclear RNAs (snRNAs) to form the corresponding core snRNPs. In this structure, strand β4 of the SmD2 protein pairs with strand β5 of the SmD1 protein [53]. One of the residues ubiquitinated by SlrP, Lys92, is included in the β4 strand. Another ubiquitinated residue, Lys85, is in loop L4 (residues 76–90). Interestingly, this is a not ordered region bearing several positively charged side chains that have been suggested to interact with a secondary structural element that is present in the majority of U snRNAs [54]. Together, these data suggest that the ubiquitination of SNRPD2 by SlrP at residues Lys85 and Lys92 may hinder the stability of spliceosomal snRNP particles. Additional experiments will be needed to test this hypothesis.

The genes encoding the NEL E3 ligases found in *S. enterica* have a complex phylogenetic distribution [55]. While *slrP* and *sspH2* are common to most salmonellae, *sspH1* has a more restricted dissemination [10] and is absent in some laboratory Typhimurium strains as well as in the better characterized strains of typhoidal *Salmonella*. However, the simultaneous presence of two or more effectors of this family in the same bacteria raises questions about the specificity and redundancy of the activity of E3 ligases toward particular substrates. For example, strain 14,028 of *S. enterica* serovar Typhimurium, used in our experiments, expresses SlrP, SspH1, and SspH2. These three proteins have similar architectures, with an N-terminus involved in T3SS-dependent translocation, a central LPX domain, and the catalytic C-terminal NEL domain [15]. The LPX domains are supposed to be necessary for physical interactions with substrates, and previous studies indicate that they are involved in the regulation of the activity of the adjacent NEL domain [56]. In addition, our previous structural studies of SlrP demonstrated that the linker region between the LPX and NEL domains plays an essential role in substrate binding [27]. The LPX domains of the three effectors contain a different number of LRR motifs: 10 for SlrP, 8 for SspH1, and 12 for SspH2 [15], and BLASTP comparisons indicate that while SspH1 and SspH2 are 62% identical, the overall sequence identity of SlrP with the other two members of the family is about 40%. The results presented here indicate that SlrP was the only one able to interact with SNRPD2 using the two-hybrid system, although some interaction was also observed for SspH1 in a pull-down experiment, suggesting a weaker in vitro interaction of SNRD2 with this effector. More importantly, SNRPD2 was specifically ubiquitinated in the presence of SlrP, but not in the presence of SspH1 or SspH2. These results support the idea that these proteins, in spite of their similar domain composition and catalytic activity, have specific host targets and might manipulate different host processes during infection.

## 5. Conclusions

In this work, we carried out a genetic screen to identify new targets for the *Salmonella* effector SlrP. A total of fourteen new interacting partners were identified and, among them, the splicing factor SNRPD2 was shown to be a specific substrate for the E3 ubiquitin ligase activity of SlrP.

The identification of host targets for *Salmonella* T3SS effectors is the first step in understanding the cellular processes that are manipulated by these virulence molecules. In fact, very few catalytic substrates have been identified for the three *Salmonella* members of the NEL family: PKN1 for SspH1 [57], NOD1 for SspH2 [58], and thioredoxin for SlrP [17]. The identification of SNRPD2 as a new target for SlrP ubiquitination is a relevant contribution to understanding the role of this *Salmonella* effector and opens new perspectives to investigate how this fascinating intracellular pathogen hijacks essential host functions such as mRNA metabolism.

## Figures and Tables

**Table 1:** Microbial strains and plasmids used in this study.

Strain/Plasmid	Relevant Characteristics	Source/Reference
***Escherichia coli***		
BL21(DE3)	F^-^ *ompT gal dcm lon hsdS_B_* (r^-^ m^-^; *E. coli* B strain), with DE3, a λ prophage carrying the T7 RNA *pol* gene	Stratagene
DH5α	*supE44 ∆lacU*169 (Ø80 *lacZ*∆M15) *hsdR17 recA1 endA1 gyrA96 thi-1 relA1*	[21]
HB101	*F^-^ mcrB mrr hsdS20 (r_B_^-^ m_B_^-^) recA13 leuB6 ara-14 proA2 lacY1 galK2 xyl-5 mtl-1 rpsL20*(Sm^R^) *glnV44λ^-^*	[22]
M15	*lac ara gal mtl*	
XL1-Blue	*recA1 endA1 gyrA96 thi-1 hsdR17 supE44 relA1 ∆lac-pro/F’ proAB lacI^q^ lacZ∆M15 Tn10* (Tet^r^)	[23]
***Salmonella enterica* serovar Typhimurium^a^**		
14028	Wild type	ATCC
SV5193	*slrP*::3xFLAG, Km^r^	[17]
***Saccharomyces cerevisiae***		
L40	*MATα trp1 leu2 his3 LYS2::lexA-HIS3 URA3::lexA-lacZ*	[24]
***Plasmids***		
pCS2-3xHA	Mammalian expression vector	Laboratory stock
pGEX-4T-1	GST fusion vector, Ap^r^	GE Healthcare
pGEX-4T-2	GST fusion vector, Ap^r^	GE Healthcare
pGAD1318	Yeast two-hybrid vector, Ap^r^	[25]
pLEX10	Yeast two-hybrid vector, Ap^r^	[26]
pIZ1628	pLEX10-SlrP	This work
pIZ1720	pCS2-SlrP-3xFLAG	[17]
pIZ1725	pcDNA3-SlrP-3xFLAG	[17]
pIZ1749	pQE30-SlrP	This work
pIZ1784	pQE30-SlrP(140-765)	[27]
pIZ2177	pQE80L-SseK1	Laboratory stock
pIZ2370	pGAD1318-SNRPD2	This work
pIZ3403	pGEX-4T-2-SNRPD2	This work
pIZ3407	pLEX10-SspH1	This work
pIZ3408	pLEX10-SspH2	This work
pIZ3542	pQE80L-SlrP(C546A)	This work
pIZ3551	pCS2-3HA-SNRPD2	This work
pIZ3557	pGEX-4T-2-SNRPD2(K85A)	This work
pIZ3558	pGEX-4T-2-SNRPD2(K92A)	This work
pIZ3562	pGEX-4T-2-SNRPD2(K85A/K92A)	This work
pIZ3591	pGEX-4T-2-SNRPD2(∆84-92)	This work
pIZ3597	pQE80L-SspH1	This work
pIZ3598	pQE80L-SspH2	This work
pQE80L	6His fusion vector, Ap^r^	Qiagen
pREP4	*lacI* Km^r^	Qiagen

**Table 2:** Oligonucleotides used in this study.

Oligonucleotide/Use	Sequence 5′-3′
**Construction of pIZ3407**	
sspH1bamfw	ATGCGGATCCATGTTTAATATCCGCAATAC
sspH1xhorv	TGACCTCGAGTCAGTTAAGACGCCACCGGG
**Construction of pIZ3408**	
sspH2ecofw	ATGCGAATTCATGCCCTTTCATATTGGAAG
sspH2salrv	GATCGTCGACTCAGTTACGACGCCACTGAAC
**Construction of pIZ3509**	
TBCBecoRIfw	ATCGGAATTCGAGGTGACGGGGGTGTCGGC
TBCBSTOPxhoIrev	ATCGCTCGAGGTCATATCTCGTCCAACCCG
**Construction of pIZ3551**	
SNRPD2ecofw	ATGCGAATTCAGCCTCCTCAACAAGCCCAAG
SNRPD2bamrv	ATCGTCTAGACTACTTGCCGGCGATGAGC
**Construction of pIZ3557**	
SNRPD2K85Afw	GTGGCAAGGGCAAGGCGAAGTCCAAGCCAG
SNRPD2K85Arv	CTGGCTTGGACTTCGCCTTGCCCTTGCCAC
**Construction of pIZ3558**	
SNRPD2K92Afw	CCAAGCCAGTCAACGCAGACCGCTACATCTC
SNRPD2K92Arv	GAGATGTAGCGGTCTGCGTTGACTGGCTTGG
**Construction of pIZ3591**	
SNRPD2-84-92delfw	CAAGAGTGGCAAGGGCGACCGCTACATCTCC
SNRPD2-84-92delrv	GGAGATGTAGCGGTCGCCCTTGCCACTCTTG
**Amplification of pQE80L**	
pQE80fw	CTGAGCTTGGACTCCTGTTG
pQE80rev	GTGATGGTGATGGTGATGCG
**Construction of pIZ3597**	
P1-pQE80-sspH1-fw	CACCATCACCATCACATGTTTAATATCCGCAATACACAACC
P2-pQE80-sspH1-rv	GGAGTCCAAGCTCAGTCAGTTAAGACGCCACCGGG
**Construction of pIZ3598**	
P1-pQE80-sspH2-fw	CACCATCACCATCACATGCCCTTTCATATTGGAAGC
P2-pQE80-sspH2-rv	GGAGTCCAAGCTCAGTCAGTTACGACGCCACTGAAC
**Checking of SNRPD2 mutations**	
SNRPD2bamHIfw	GATCGGATCCATGAGCCTCCTCAACAAGCC
SNRPD2comp-K85A-rv	GTTGACTGGCTTGGACTTCGC
SNRPD2comp-K92A-rv	CTTGGAGATGTAGCGGTCTGC
SNRPD2comp-Del84-92-rv	CTTTGTTGACTGGCTTGGAC
**Identification of candidates carrying LSM2**	
LSM2fw	TCAAGTCCCTTGTGGGCAAG
LSM2rev	TCACTGTTTCTGCTGCAGGG
**Identification of candidates carrying PPP1R7**	
PPP1R7fw	CTAAACTTCAGAACCTGGATG
PPP1R7rev	TCAGAACCTGACGAACGTGG
**Identification of candidates carrying RABIF**	
RABIFfw	CGTTGCGGCTCCCGGGTGCTG
RABIFrev	TTACTCATGGGAAACTCGTTC
**Identification of candidates carrying SNRPD2**	
SNRPD2fw	AGGAGCTGCAGAAGCGAGAG
SNRPD2rev	CTACTTGCCGGCGATGAGCG
**Identification of candidates carrying TRX**	
tio5′	GTCAGAATTCGCCGCCACGATGGTGAAGCAGATC
tio3′	GTCAGAATTCGCCGCCACGATGGTGAAGCAGATC
**Sequencing of two-hybrid screen candidates**	
Gal4AD	TACCACTACAATGGATG

**Table 3:** Candidate host partners of SlrP identified in a yeast two-hybrid screen.

Gene	Number of Clones	Description of the Product	Amino Acids Encoded in Different Clones ^1^
ABHD14B	5	Serine hydrolase with lysine deacetylase activity	1–210
ANP32A	2	Acidic leucine-rich nuclear phosphoprotein	25–249
CEP97	5	Centrosomal protein	120–865
EXOSC7	4	Exosome complex component	40–291
LSM2	29	Sm-like protein with a role in pre-mRNA splicing	1–95/6–95
MITD1	9	Required for efficient abscission at the end of cytokinesis	1–249/8–249/72–249
NOP58	4	Nucleolar protein required for 60S ribosomal subunit biogenesis	360–529
PLK4	11	Serine/threonine protein kinase	698–970/727–970/777–970/837–970
PPP1R7	9	Regulatory subunit of protein phosphatase 1	196–360/203–360
RABIF	8	Guanine nucleotide exchange factor	1–123/4–123
SNRPD2	236	Small nuclear ribonucleoprotein with a role in pre-mRNA splicing	1–118/10–118/13–118/15–118/19–118
TBCE	4	Tubulin-folding protein	129–527
TXN	220	Thioredoxin	1–105
XRCC6	6	Single-stranded DNA-dependent ATP-dependent helicase	315–609/402–609/405–609/464–609
ZFPM1	3	Zinc finger protein	895–1006

^1.^ C-terminal end always included.

## Data Availability

The data that support the findings of this study are available from the corresponding author upon reasonable request.
